# Knowledge, Attitude, and Practices Regarding the Rome IV Criteria for the Diagnosis of Irritable Bowel Syndrome Among Primary Healthcare Physicians in Saudi Arabia

**DOI:** 10.7759/cureus.49460

**Published:** 2023-11-26

**Authors:** Tahani A Khalil, Doaa Mohammad Filmban, Hussam Nasser M Sinnah, Rahma Abdullah Fallatah, Asmaa A Sayis, Sarah S Alruwaili, Sultan A Alghamdi, Nura Fahad M Almutairi, Alyaa Mohammed I Haider, Razan A Ajam, Abeer F Basmih

**Affiliations:** 1 Family Medicine, King Salman Armed Forces Hospital, Tabuk, SAU; 2 Internal Medicine, Al-Noor Specialist Hospital, Mecca, SAU; 3 Research and Development, King Khalid University, Abha, SAU; 4 Internal Medicine, Al-Haram Hospital, Medina, SAU; 5 Faculty of Medicine, Al Jouf University, Sakakah, SAU; 6 Faculty of Medicine, King Abdulaziz University, Jeddah, SAU; 7 Faculty of Medicine, University of Tabuk, Tabuk, SAU; 8 Faculty of Medicine, Ibn Sina National College, Jeddah, SAU

**Keywords:** primary healthcare physicians, saudi arabia, practice, attitude, knowledge, irritable bowel syndrome

## Abstract

Introduction: Irritable bowel syndrome (IBS) presents a significant challenge in the medical field due to its complex nature as a functional gastrointestinal illness (FGID) without clear biological markers. Diagnosis often involves ruling out other potential causes, leading to frustration for patients and difficulty in effective treatment. Given its high prevalence among FGIDs, primary healthcare (PHC) physicians play a crucial role in its initial assessment and management.

Methods: This cross-sectional study surveyed 623 PHC physicians using a structured, closed-ended questionnaire. Employing a multistage sampling approach, physicians were selected from various provinces and cities in Saudi Arabia. Clusters within these cities were also sampled.

Results: The research revealed that a majority of physicians (n = 411, 66%) exhibited a strong awareness of the Rome IV criteria, essential for diagnosing IBS. This awareness correlated significantly with variables, such as age, nationality, marital status, specialty, classifications, and years of experience.

Conclusion: PHC physicians in the study demonstrated a commendable level of familiarity with the Rome IV criteria for diagnosing IBS. Their knowledge was particularly robust concerning symptoms outlined in these criteria. However, there is room for improvement in their understanding, attitude, and application of the Rome IV guidelines in practice. Addressing these gaps could enhance the overall management of IBS cases by these physicians.

## Introduction

Irritable bowel syndrome (IBS), a common functional gastrointestinal disorder (FGID), is characterized by recurrent episodes of stomach pain or discomfort. Its prevalence varies globally, with estimates ranging from 10% to 25% in the US and significant regional differences, notably in South America (17-21%), South Asia (7-9%), and the Middle East and Africa (5.6%). Approximately 40% of people meet the criteria for FGID, including IBS, with women experiencing 1.5 to three times as many symptoms as men. IBS affects individuals of all ages, with 50% of those under 35 reporting symptoms; however, prevalence declines beyond age 50 [1,2].

Globally, IBS prevalence ranges from 10% to 23%, with first-degree relatives being more susceptible [3]. Studies in diverse settings have shown varying IBS rates in Saudi Arabia, ranging from 8% to 40%. Alarmingly, between 33% and 90% of patients do not seek adequate medical help, posing a significant public health risk. In addition, IBS is linked to mental health issues, specifically anxiety and depression, which can exacerbate symptoms due to stress in the large intestine [4].

IBS is a complex illness influenced by factors, such as changes in gut microbiota, visceral hypersensitivity, inflammation, gastrointestinal dysmotility, stress exposure (especially in early life), and diet. The autonomic nervous system (ANS) and the hypothalamic-pituitary-adrenal (HPA) axis, key players in the stress response system, are often studied in IBS research. Genetic predisposition and environmental interactions, including familial susceptibility and psychosocial stressors, also contribute to the condition's pathophysiology [5,6].

IBS significantly impacts life quality and is closely associated with mental health issues, making it a serious public health concern. Despite its usually benign prognosis, IBS can mask more severe conditions, such as colon cancer, emphasizing the need for accurate diagnosis. Diagnosing IBS is challenging due to the absence of specific biochemical or biological indicators and the individual variation in symptom presentation [7,8].

Clinical criteria, including the Manning criteria and Rome IV criteria, are used for diagnosis. The Rome IV criteria include persistent stomach ache for at least three months, occurring at least twice a week, and associated with two or more of the following: (i) related to bowel movements, (ii) changes in frequency, or (iii) changes in stool consistency [9]. Primary healthcare (PHC) practitioners can diagnose IBS effectively by considering the patient's medical history and using the Rome IV criteria, which are widely regarded as the gold standard for diagnosing IBS in clinical settings [10].

It has been proposed that the effective identification and diagnosis of IBS patients can be achieved through a primary care approach by screening those individuals who frequently visit healthcare facilities [11]. Recognizing the crucial role of PHC physicians in managing chronic gastrointestinal disorders, including IBS, has gained prominence in recent times [12].

The utilization of the Rome IV criteria for identifying and diagnosing IBS is considered a straightforward and effective method, relying on patient history, making it easily implementable by PHC physicians [13]. Unfortunately, studies have revealed that the knowledge and application of this diagnostic tool among PHC physicians are not at a satisfactory level [14].

While some studies on IBS prevalence in Saudi Arabia focused on specific groups, such as school teachers and college students, using the earlier Rome criteria, there is limited information on the condition's prevalence in the general population [14-17]. Recent studies employing the Rome IV criteria have indicated a prevalence of 16.3% among participants, highlighting the need for more comprehensive research [18].

Despite numerous global studies on IBS, there is a lack of research assessing physicians' knowledge, particularly in Saudi Arabia, about the illness and its symptoms. Therefore, we conducted a study to evaluate the knowledge, attitudes, and practices of PHC physicians in our region regarding the Rome IV criteria for diagnosing IBS. This study aims to fill the existing gap in understanding and improve the diagnosis and management of IBS, ultimately enhancing patient care and public health outcomes.

## Materials and methods

Study design 

This cross-sectional study aimed to assess the knowledge, attitudes, and practices of PHC physicians regarding the Rome IV criteria for the diagnosis of IBS.

Study area

The research was conducted across various provinces and cities in Saudi Arabia, covering 13 regions: Riyadh, Buraidah, Dammam, Ha'il, Al-Baha, Mecca, Medina, Arar, Abha, Najran, Jazan, Tabuk, and Sakaka.

 Study population

The study included active PHC physicians in Saudi Arabia, comprising general practitioners, residents, specialists, or consultants, engaged in clinical practice and willing participants.

Inclusion and exclusion criteria

The inclusion criteria included active PHC physicians, including general practitioners, residents, specialists, or consultants, physicians currently employed and actively engaged in clinical practice, and participants who provided informed consent.

The exclusion criteria are PHC physicians not currently employed and physicians unwilling to participate.

Sample size

In a study conducted in 2020, it was reported that there were approximately 6,107 PHC physicians in Saudi Arabia [19]. To determine the sample size for our study, we utilized the Raosoft sample size calculator (Raosoft Inc., Seattle, WA, USA, raosoft.com). Calculations indicated that 362 participants were required to achieve a 95% confidence interval and a 5% margin of error, assuming a 50% prevalence rate.

We employed a proportional stratified random sampling technique based on regions/cities to determine the number of participants included in this study. This method ensured representation from various regions and cities after data collection.

Sampling technique

Stage 1: Selection of Provinces

The initial phase of the sampling process involved choosing representative provinces in Saudi Arabia, aiming to encompass the country's geographical diversity and healthcare infrastructure. A stratified sampling technique was employed to guarantee the inclusion of various provinces, ensuring a balanced representation.

Stage 2: Selection of PHC Centers

Within each chosen province, a random sampling technique was applied to identify PHC centers. The number of centers selected from each province was proportionate to the total count of PHC centers in that area. This approach ensured the inclusion of centers from diverse provinces, contributing to the sample's comprehensiveness.

Stage 3: Selection of PHC Physicians

In each selected PHC center, a random sampling method was used to select PHC physicians. A suitable number of physicians from different regions of Saudi Arabia were included, emphasizing diversity and representation. This methodology aimed to guarantee the sample's representativeness, providing an adequate number of participants for the study.

Study variables

The study encompassed demographic details, along with assessing knowledge, attitudes, and practices of PHC physicians concerning the Rome IV criteria for diagnosing IBS.

Data collection tools

The data were collected by multiple data collectors using a self-administered questionnaire. The questionnaire was designed on online survey platforms and distributed to PHC physicians through random sampling interviews. The survey was modified from a previously published version [8].

The questionnaire consisted of two main parts. The first section covered sociodemographic factors, such as age, gender, nationality, marital status, specialty, physician level, years of practice, and monthly income. The second part comprised questions to assess general awareness of the Rome IV criteria (three questions), knowledge (six questions), attitude (two questions), and practice (five questions) related to the diagnosis of IBS. However, participants who had not heard of the Rome IV criteria were excluded from answering the remaining questions. Similarly, physicians who had not used the Rome IV criteria were excluded from answering the practice-related questions.

The Rome IV criteria, widely accepted for diagnosing IBS, specified that symptoms must have persisted for at least six months, with regular abdominal pain occurring at least once per day or once per week in the previous three months. In addition, at least two symptoms related to defecation and altered stools in terms of frequency, form, or appearance must have been present [20].

To ensure clarity and understanding, a pilot study was conducted among 15 PHC physicians who were not part of the main study. This pilot assessed the questionnaire's comprehensibility and the time required to complete it.

Data analysis plan

IBM SPSS Statistics for Windows, version 28 (released 2021; IBM Corp., Armonk, New York, United States) was used for data analysis. Descriptive statistics, including frequencies, percentages, averages, and standard deviations, were employed. The chi-square test was used to assess associations between sociodemographic variables and awareness of the Rome IV criteria. Statistically significant differences were determined at a p-value of ≤0.05.

Ethical consideration

Ethical clearance for this study was granted by the Institutional Review Board (IRB) of King Salman Armed Forces Hospital, with approval number KSAFH-REC-2023-525. Prior to their involvement in the trial, all participants provided oral informed consent. Rigorous measures were in place to ensure the confidentiality and privacy of all participants.

## Results

In Table 1, the age group with the highest frequency was 20 to 30 years old (n = 339, 54.4%). Males constituted the majority in gender (n = 334, 53.6%), and Saudi nationals represented the highest frequency in nationality (n = 526, 84.4%). Most participants were general practitioners (n = 270, 43.3%), and resident physicians had the highest frequency in terms of classification (n = 380, 61%). In addition, the majority of participants had less than three years of experience (n = 312, 50.1%).

**Table 1:** Sociodemographic information (n = 623) N: number of participants, %: percentage of participants

Variables	Classifications	N	%
Age	20-30 years	339	54.4%
	31-40 years	193	31%
	>40 years	91	14.6%
Gender	Male	334	53.6
	Female	289	46.4
Nationality	Saudi	526	84.4
	Non-Saudi	97	15.6
Marital status	Been married	248	39.8
	Never been married	375	60.2
Specialty	General practitioners	270	43.3
	Family medicine	116	18.6
	Others	237	38.0
Classification	Resident	380	61.0
	Specialist	148	23.8
	Consultant	95	15.2
Years of practice	<3 years	312	50.1
	3-5 years	201	32.3
	>5 years	110	17.7

Table 2 reveals that the age group between 20 and 30 years old exhibited the highest awareness among various age groups (n = 182, 53.7%). The p-value was highly significant (<0.0001). Both females (n = 193, 66.8%) and males (n = 218, 65.3%) demonstrated nearly equal awareness (p < 0.05, non-significant). Saudi nationals exhibited higher awareness (n = 359, 68.3%). The p-value was significant (<0.0001), attributed to the higher frequency of Saudi nationals compared to non-Saudi individuals. Married participants displayed a significantly higher awareness (n = 197, 79.4%) compared to those who had never been married (p ≤ 0.0001, significant). Family medicine physicians exhibited higher awareness (n = 101, 87.1%), which was highly significant (p ≤ 0.0001). Similarly, consultant physicians (n = 83, 87.4%) and physicians with three to five years of practice (n = 169, 84.1%) demonstrated higher awareness, both being highly significant (p ≤ 0.0001).

**Table 2:** Association of awareness of the Rome IV criteria among the studied participants regarding the demographic characters (n = 623) * Fisher’s exact test

Have you heard about the Rome IV criteria?				
Variables	Classes	Yes (411, 66%)	No (212, 34%)	p-value
Age group	20-30 years	182 (53.7%)	157 (46.3%)	<0.0001
	31-40 years	156 (80.8%)	37 (19.2%)	
	>40 years	73 (80.2%)	18 (19.2%)	
Gender	Male	218 (65.3%)	116 (34.7%)	<0.378*
	Female	193 (66.8%)	96 (33.2%)	
Nationality	Saudi	359 (68.3%)	167 (31.7%)	<0.004*
	Non-Saudi	52 (53.6%)	45 (46.4%)	
Marital status	Been married	197 (79.4%)	51 (20.6%)	<0.0001*
	Never been married	214 (57.1%)	161 (42.9%)	
Specialty	General practitioner	164 (60.7%)	106 (39.3%)	<0.0001
	Family medicine	101 (87.1%)	15 (12.9%)	
	Others	146 (61.6%)	91 (38.4%)	
Classification	Resident	210 (55.3%)	170 (44.7%)	<0.0001
	Specialist	118 (79.7%)	30 (20.3%)	
	Consultant	83 (87.4%)	12 (12.6%)	
Years of practice	<3 years	157 (50.3%)	155 (49.7%)	<0.0001
	3–5 years	169 (84.1%)	32 (15.9%)	
	>5 years	85 (77.3%)	25 (22.7%)	

As shown in Table 3, the participants demonstrated higher knowledge about the symptoms of IBS. Recurrent abdominal pain was the most commonly recognized symptom (n = 511, 82.02%). However, when asked about the symptoms preceding the application of the Rome IV criteria, the participants' responses were insufficient. The highest response was "more than six months" (n = 316, 50.7%). Similarly, when questioned about the duration the criteria should be met, the participants' responses were inadequate. The maximum response (n= 250, 40.1%) was "three months."

**Table 3:** Knowledge about the components of the Rome IV criteria N: number of participants, %: percentage of participants

Variables	Classifications	N	%
Symptoms of IBS or functional bowel disorder that are addressed in the Rome IV criteria	Associated with change in stool appearance	423	67.90
	Associated with change in stool frequency	432	69.34
	Recurrent abdominal pain	511	82.02
	Relation of abdominal pain to defecation	453	72.71
The symptom onset should be for how much duration to apply the Rome IV criteria.	<6 months	103	16.5
	>6 months	316	50.7
	I don’t know	140	22.5
	No relation	64	10.3
The criteria should be fulfilled for how much of the time duration.	1 month	59	9.5
	2 months	63	10.1
	3 months	250	40.1
	6 months	100	16.1
	I don’t know	151	24.2

According to Table 4, the majority of participants noted that between 25% and 50% of patients met the eligibility requirements for applying the Rome IV criteria (n = 236, 37.9%). In addition, a significant portion (n = 170, 27.3%) of patients lacked information in this regard. Approximately half of the participants also indicated that they believed the Rome IV criteria were sufficient for diagnosing IBS.

**Table 4:** Attitude toward the Rome IV criteria for diagnosing IBS N: number of participants, %: percentage of participants

Variables	Classifications	N	%
In your opinion, what proportion of the patients qualify for the Rome IV criteria to be applied for diagnosing IBS?	<25%	114	18.3
	25–50%	236	37.9
	>50	103	16.5
	I don't know	170	27.3
Do you feel that the Rome IV criteria are effective enough to diagnose IBS?	Yes	310	49.8
	No	124	19.9
	I don't know	189	30.3

Table 5 indicates that the participants' responses concerning the use of the Rome IV criteria were mixed. Of the participants, (n = 322, 51.7%) stated "yes," while the remaining (n= 301, 48.3%) said "no." Regarding the frequency of using the Rome IV criteria, 217 (34.8%) did not use it at all, while 214 (34.3%) used it in selected cases and 192 (30.8%) participants used it for all cases. The participants' responses regarding the cases of IBS that could be referred to specialists were also varied. Regarding the ability to achieve continuity of care for IBS, the responses showed that 174 (37.9%) always achieved continuity of care, while the majority (n = 323, 51.8%) were only sometimes able to do so. Concerning participation in raising awareness, the majority of participants responded positively (n = 445, 71.4%).

**Table 5:** Practice toward the Rome IV criteria for diagnosing IBS N: number of participants, %: percentage of participants

Variables	Classifications	N	%
Have you ever used the Rome IV criteria to diagnose IBS?	Yes	322	51.7
	No	301	48.3
Do you frequently use the Rome IV criteria to diagnose IBS?	Don’t use it at all	217	34.80
	For selected cases	214	34.30
	Yes, for all cases	192	30.80
Which cases of IBS do you consider for specialist referral?	All patients	138	22.2
	Development of complications	180	28.9
	Long duration of patients	210	33.7
	None	95	15.2
Are you able to achieve continuity of care for IBS patients?	Always	174	27.9
	Sometimes	323	51.8
	Rarely	70	11.2
	Never	56	9
Have you participated in raising awareness?	Yes	445	71.4
	No	178	28.6

In Table 6, it can be observed that there was no significant difference (p = 0.378) between the sexes regarding awareness of the Rome IV criteria (females: n = 193, 66.8%; males: n = 218, 65.3%). However, a highly significant difference (p = 0.0001) was noted between the sexes concerning their knowledge of IBS symptoms, with males demonstrating higher awareness than females. Regarding the duration of symptom onset, no significant difference was found between the sexes (p = 0.978). Notably, a highly significant difference in the knowledge of the criteria was observed between genders (p = 0.0001), with males exhibiting higher awareness compared to females. When participants were asked about the percentage of patients meeting the requirements for using the Rome IV criteria to diagnose IBS, there was no significant difference between males and females (p = 0.246). However, a significant difference was observed when the participants were asked if they believed that the criteria were sufficient for diagnosing IBS (p = 0.009), with males exhibiting a more positive attitude than females. Regarding the use of the Rome IV criteria to diagnose IBS, women responded more positively than men (p = 0.003). A significant difference was noted in the frequency of using the criteria to diagnose IBS between the genders (p = 0.046). However, no significant differences were found between genders in considering IBS cases for specialist referral (p = 0.921), ensuring continuity of care for IBS patients (p = 0.295), and participating in awareness-raising efforts (p = 0.666).

**Table 6:** Association between the participants' knowledge, attitude, and practices with the components of the Rome IV criteria regarding gender. * Fisher’s exact test (sig.)

Variables	Responses of the participants	Female	Male	p-value
		N = 289 (46.4%)	N = 334 (53.6%)	
Have you heard about the Rome IV criteria?	Yes	193 (66.8%)	218 (65.3%)	<0.378
	No	96 (33.2%)	116 (34.7%)	
Symptoms of IBS or functional bowel disorder that are addressed in the Rome IV criteria	Associated with change in stool appearance	164 (49.10%)	209 (62.57%)	<0.0001
	Associated with change in stool frequency	185 (55.39%)	247 (73.95%)	
	Recurrent abdominal pain	194 (58.02%)	268 (80.24%)	
	Relation of abdominal pain to defecation	204 (61.08%)	249 (74.55%)	
The symptom onset should be for how much duration to apply the Rome IV criteria.	<6 months	47 (16.30%)	56 (16.80%)	<0.978
	>6 months	145 (50.20%)	171 (51.20%)	
	I don’t know	66 (22.80%)	74 (22.20%)	
	No relation	31 (10.7%)	33 (9.90%)	
The criteria should be fulfilled for how much of the time duration.	1 month	34 (11.80%)	25 (7.50%)	<0.0001
	2 months	22 (7.60%)	41 (12.30%)	
	3 months	98 (33.90%)	152 (45.50%)	
	6 months	68 (23.50%)	32 (9.60%)	
	I don’t know	67 (23.20%)	84 (25.10%)	
In your opinion, what proportion of patients qualify for the Rome IV criteria to be applied for diagnosing IBS?	<25%	62 (21.5%)	52 (15.60%)	<0.246
	25–50%	107 (37.0%)	129 (38.60%)	
	>50	48 (16.60%)	55 (16.50%)	
	I don't know	72 (24.90%)	98 (29.30%)	
Do you feel that the Rome IV criteria are effective enough to diagnose IBS?	Yes	140 (48.40%)	170 (50.9%)	<0.009
	No	72 (24.90%)	52 (15.6%)	
	I don't know	77 (26.60%)	112 (33.5%)	
Have you ever used the Rome IV criteria to diagnose IBS?	Yes	167 (57.80%)	155 (46.40%)	0.003*
	No	122 (42.20%)	179 (53.60%)	
Do you frequently use the Rome IV criteria to diagnose IBS?	Don’t use it at all	86 (29.80%)	131 (39.20%)	<0.046
	For selected cases	108 (37.40%)	106 (31.70%)	
	Yes, for all cases	95 (32.90%)	97 (29.00%)	
Which cases of IBS do you consider for specialist referral?	All patients	63 (21.80%)	75 (22.50%)	<0.921
	Development of complications	87 (30.10%)	93 (27.80%)	
	Long duration of patients	97 (33.60%)	113 (33.80%)	
	None	42 (14.50%)	53 (15.90%)	
Are you able to achieve continuity of care for IBS patients?	Always	91 (31.50%)	83 (24.90%)	<0.295
	Sometimes	145 (50.20%)	178 (53.30%)	
	Rarely	29 (10.0%)	41 (12.30%)	
	Never	24 (8.30%)	32 (9.60%)	
Have you participated in raising awareness?	Yes	204 (70.60%)	241 (72.20%)	<0.666
	No	85 (29.40%)	93 (27.80%)	

## Discussion

The symptoms of IBS, a chronic functional bowel condition that frequently recurs, include diarrhea, constipation, bloating, cramping, and abdominal pain [19]. These gastrointestinal symptoms are caused by an illness that affects both the small and large intestines. IBS is termed a "syndrome" because it comprises a collection of symptoms that irritate the stomach; however, each person experiences these symptoms differently [20].

Given the high prevalence of IBS in Saudi Arabia, there is limited information on doctors' attitudes and awareness of the illness [8]. Therefore, this study aimed to assess the self-reported knowledge, attitudes, and practices related to the Rome IV criteria for diagnosing IBS among PHC physicians in various regions and cities of Saudi Arabia.

Regarding the demographic characteristics of the participants, the age group from 20 to 30 years old had the highest frequency (n = 339, 54.4%). Males constituted the majority in gender representation (n = 334, 53.6%). Saudi nationality was predominant among the participants (n = 526, 84.4%). The majority of participants were general practitioners (n = 270, 43.3%), while resident physicians represented a significant portion (n = 380, 61%). Participants with less than three years of experience were the most frequent (n = 312, 50.1%). The age and gender in our study were on the same line as reported in Hungin et al.'s study [21], and the studied general practitioners were predominant in some other studies as in Abusageah et al.'s study [8], which supported our findings.

The awareness level in our study was relatively high, with about two-thirds of the participants (n = 411, 66%) demonstrating awareness. In a similar study conducted in Riyadh, the awareness level was reported to be 86% [4], and in another study in Jazan, it was 78% [8]. Both of these studies showed higher awareness levels compared to our results. This difference could be attributed to the smaller sample size in the previous studies; for instance, the Riyadh study had 216 participants, and the Jazan study had 200 participants. Another reason is that the participants in the study in Riyadh [4] and Jazan [8] were selected randomly from particular centers, unlike our study where we included all the participants. Despite these variations, our research demonstrated a higher level of awareness compared to some other studies. For example, a study conducted in Iceland reported an awareness level of 65% [22], and another study by Hungin et al. found awareness levels ranging from 2% to 36% among the participants [21].

In our research, as indicated in Table 3, the participants exhibited a higher level of knowledge regarding the Rome IV criteria, particularly concerning the symptoms of IBS. The most common symptom reported was recurrent abdominal pain, identified by 511 participants, accounting for 82.02% of the responses. These findings align with the results of a study conducted by McOmber et al. [23]. However, regarding the duration of symptoms before applying the Rome IV criteria, the participants provided insufficient answers. The majority indicated a duration of more than six months (n = 316, 50.7%). This result was consistent with another previous study that supported our findings [24].

In Table 4, the attitudes of the participants in our research were analyzed. The majority of the participants (n = 236, 37.9%) believed that 25-50% of patients were eligible for applying the Rome IV criteria. These findings were consistent with the results of Black and Ford's study [25]. In addition, 27.3% of participants (n = 170) did not have sufficient information on this matter. Furthermore, almost 50% of our participants considered the Rome IV criteria effective for diagnosing IBS. This contrasts with another study where 70% of participants believed in the effectiveness of the Rome IV criteria for diagnosing IBS. The variation in these results could be attributed to the differences in the number of participants between the two studies [8].

As shown in Table 5, in our study, 192 participants (30.8%) showed frequently using the Rome IV criteria in all cases, while 214 (34.3%) used it in selective cases; these results were higher than a study conducted in Jazan in 2022, which included 200 participants, where 56 (28%) of the physicians reported using the Rome IV criteria frequently in their daily practice and 61 physicians (30.5%) used it in selected cases [8]. Our findings also differed from those reported by Al-Hazmi et al. [26], whose results indicated a usage rate of 29.4%, and Al-Shamrani et al. [4], who reported a usage rate of 23% among physicians employing the Rome IV criteria. Specifically, 217 participants (34.8%) did not use the Rome IV criteria at all, 214 participants (34.3%) used it in selected cases, and 192 participants (30.8%) used it for all cases. Regarding the referral of IBS cases to specialists, the responses provided were inadequate. Our findings align with the study conducted by Vasant et al., which recommended specialist review for patients with severe symptoms [27]. Regarding the ability to achieve continuity of care for IBS patients, our participants' responses indicated that 174 participants (37.9%) always achieved continuity of care, while the majority (n = 323, 51.8%) sometimes achieved continuity. By contrast, a study by Al-Shamrani et al. [4] found that 43% of physicians actively participated in increasing awareness. However, our study showed that the majority of participants had a positive response toward raising awareness, with 445 participants (71.4%) indicating their willingness to participate in awareness-raising activities.

Table 6 presents the association among participants' awareness, knowledge, attitude, and practices related to the components of the Rome IV criteria, stratified by gender. Statistical analysis showed no significant difference between females and males (p = 0.378) concerning awareness about the Rome IV criteria. Specifically, 66.8% of females (n = 193) and 65.3% of males (n = 218) demonstrated awareness about the symptoms of IBS. These results align with findings from previous studies [4,8].

The study conducted in Jazan [8] and Riyadh [4] revealed that there was no significant difference in knowledge between males and females concerning the application of the Rome IV criteria for diagnosing IBS (p = 0.457 and p = 0.430, respectively). By contrast, a highly significant difference was observed between genders (p = 0.0001), indicating that males possessed superior knowledge compared to females regarding the appropriate duration for symptom onset. Regarding the belief about the percentage of patients meeting the requirements for using the Rome IV criteria to diagnose IBS, there was no significant difference in responses between males and females (p = 0.246). However, a significant gender difference emerged when respondents were asked if they believed the criteria were sufficient to diagnose IBS (p = 0.009), with males displaying a more positive attitude than females. In terms of practical application, women exhibited a more positive response than men when asked if they had ever used the Rome IV criteria to diagnose IBS (74% vs. 70%), and this difference was statistically significant (p = 0.003). These findings align with the results of the study conducted by Saito et al. [28].

Limitations of the study

The study, while being the first of its kind to explore IBS-related knowledge and risk factors among PHC physicians across multiple provinces in Saudi Arabia, does have several limitations. First, due to its observational nature, the study inherently carries limitations typical of this research design. Second, the utilization of a self-administered questionnaire raises concerns about the authenticity of the responses provided. Furthermore, the study did not employ a scoring system to quantify knowledge, attitude, and practice, which could have provided a more effective means of assessing the desired outcomes. These limitations should be considered when interpreting the study's findings.

## Conclusions

This study revealed a commendable level of awareness among PHC physicians regarding the Rome IV criteria for diagnosing IBS. The physicians demonstrated reliable practices, with approximately two-thirds of them frequently employing the Rome IV criteria in their daily practice to diagnose IBS, either for all cases or selected ones. Moreover, over 70% of the physicians actively participated in initiatives aimed at raising awareness about the use of the Rome IV criteria. The study also found a significant association between gender and the physicians' knowledge, attitude, and practices related to the components of the Rome IV criteria, with males exhibiting a stronger association than females. These findings highlight the positive impact of awareness initiatives while emphasizing the need for continued efforts to enhance understanding and practices among healthcare professionals, particularly focusing on addressing gender disparities in knowledge and application.
